# Incidence and Outcomes of Valve-in-Valve Transcatheter Aortic Valve Implantation in Failed Bioprosthetic Valves

**DOI:** 10.3390/jcm12185868

**Published:** 2023-09-09

**Authors:** Thorald Stolte, Jasper Boeddinghaus, Giampiero Allegra, Gregor Leibundgut, Oliver Reuthebuch, Christoph Kaiser, Christian Müller, Thomas Nestelberger

**Affiliations:** 1 Department of Cardiology, Cardiovascular Research Institute Basel (CRIB), University Hospital Basel, University of Basel, Petersgraben 4, 4031 Basel, Switzerland; 2 Department of Health Sciences and Technology, Swiss Federal Institute of Technology, 8092 Zurich, Switzerland; 3 British Heart Foundation, University Centre for Cardiovascular Science, Usher Institute, University of Edinburgh, Edinburgh EH16 4SS, UK; 4 Department of Cardiac Surgery, University Hospital Basel, University of Basel, 4031 Basel, Switzerland

**Keywords:** transcatheter aortic valve implantation, valve-in-valve, valve prosthesis degeneration

## Abstract

**Highlights:**

**What is known?**

- Transcatheter aortic valve replacement (TAVR) has become the standard of care beside sur-gical valve replacement (SAVR) in the majority of patients with aortic stenosis (AS) among all risk categories and is the preferred treatment of choice in older patients.
- Bioprosthetic valve failure is an ongoing issue necessitating reoperations or valve-in-valve interventions.
- As redo-SAVR is related to elevated peri-procedural risks and mortality, valve-in-valve in-terventions are expected to increase in the future.

**What the study adds?**

- Over the last decade, a steep increase in valve-in-valve procedures was observed, resulting in an incidence of 4% among TAVR procedures.
- Valve-in-valve interventions are feasible and showed lasting improvements in mean aortic valve gradients.
- No differences in technical and device successes or event-free survival between TAVR-in-SAVR and TAVR-in-TAVR could be observed.

**Abstract:**

Introduction: Transcatheter aortic valve replacement (TAVR) has become a widely used, comparably efficient and safe alternative to surgical aortic valve replacement (SAVR). Its utilization continues to grow, especially among younger patients. Despite improvements in durability, degeneration and subsequent re-interventions of failed prosthetic valves are still common. Even though valve-in-valve procedures have become more frequent, little is known about the trends over time or about clinical and echocardiographic long-term outcomes. Materials and Methods: Patients who underwent a valve-in-valve procedure between December 2011 and December 2022 in a large tertiary university hospital were analyzed. Primary outcomes were defined as procedural and device successes as well as event-free survival. Secondary analyses between subsets of patients divided by index valve and date of procedure were performed. Results: Among 1407 procedures, 58 (4%) were valve-in-valve interventions, with an increased frequency observed over time. Overall, technical success was achieved in 88% and device success in 85% of patients. Complications were predominantly minor, with similar success rates among TAVR-in-SAVR (TiSAVR) and TAVR-in-TAVR (TiTAVR). Notably, there were significant and lasting improvements in mean echocardiographic gradients at 1 year. Event-free survival was 76% at one month and 69% at one year. Conclusions: Over the last decade, a rising trend of valve-in-valve procedures was observed. Despite an increase in procedures, complications show a contrasting decline with improved technical and device success over time. TiSAVR and TiTAVR showed comparable rates of procedural and device success as well as similar outcomes, highlighting the utility of valve-in-valve procedures in an aging population.

## 1. Introduction

For decades, the use of bioprosthetic surgical valves has been the most frequent treatment option for patients with severe aortic stenosis (AS) [1,2]. In the last two decades, transcatheter aortic valve replacement (TAVR) has become a widely used alternative to surgical aortic valve replacement (SAVR) based on clinical trials suggesting comparable efficacy and safety profiles among older patients irrespective of risk categories [3]. In current guidelines, TAVR is recommended in patients above 65 years of age in the United States and above 75 years of age in Europe [1,2]. Recent studies have reported a significant growth of TAVR procedures in younger patients in several countries [4,5]. Despite significant improvements in design and durability in both surgical and transcatheter bioprosthetic valves, degeneration and valve failure are still common over time. Whereas re-operation of a failed bioprosthetic valve has been associated with higher periprocedural risks and mortality, the implantation of a TAVR valve into a failed valve has emerged as a less-invasive alternative [6,7].

Several studies have shown generally favorable outcomes for TAVR-in-SAVR (TiSAVR) as well as TAVR-in-TAVR (TiTAVR) procedures, with lower rates of procedural complications and significant improvement in valve function, which resulted in FDA approval for Medtronic and Edwards valves in 2015 and 2017, respectively. Several technical difficulties are associated with valve-in-valve procedures, directly related to minimal space in the aortic root (patient–prosthesis mismatch resulting in elevated gradients, valve embolization and coronary obstruction among others) [8,9,10]. Long-term data regarding clinical outcomes as well echocardiographic data are still lacking. Therefore, we aimed to show the incidence, trends and long-term outcomes of valve-in-valve procedures over a time period of eleven years in a tertiary university center using data from a prospective TAVR registry.

## 2. Methods

### 2.1. Study Design and Patient Cohort

All patients who underwent TAVR at the University Hospital Basel (USB), Switzerland, were included in a prospective national database, as part of the SwissTAVI registry, mandated by the Swiss health authorities (NCT01368250). The Swiss TAVI registry has been approved by the local cantonal ethics committee and the institutional review boards of all participating sites. All patients provided written informed consent for study participation and prospective follow-up assessment. Prior results from the study have been reported [11,12,13]. The present study included patients who underwent a valve-in-valve procedure between December 2011 and December 2022.

### 2.2. Data Collection and Clinical Endpoints

All patient-related data including baseline characteristics, procedural and follow-up information were prospectively collected and recorded in a web-based database. Clinical follow-up data were obtained through standardized interviews, documentation from referring physicians, and hospital discharge summaries. All adverse events were systematically collected and adjudicated by a dedicated clinical event committee. The outcomes of interest of the present study were based on the Valve Academic Research Consortium (VARC)-3 definitions, which were retrospectively adjudicated based on detailed documentation of reported endpoints [14].

Technical success was defined as peri-procedural success including no peri-procedural death, delivery of the device with correct positioning of a single prosthetic valve and freedom of re-intervention, surgical intervention or major access-related intervention until hospital discharge.

Device success was defined as freedom from surgery or re-intervention related to the device or to a major vascular- or access-related or cardiac structural complication and intended performance of the valve (mean gradient ≤ 20 mmHg) within 30 days in addition to technical success.

Event-free survival was based on the VARC-3 definitions and defined as survival without any of the following events: procedural or in-hospital death, coronary occlusion, stroke, permanent pacemaker implantation, myocardial infarction, major (stent or surgery) vascular complications, major bleeding requiring more than two transfusions and valve embolization [14]. Secondary analyses were performed for comparison between TiSAVR and TiTAVR and between an early and late procedure using the mean of all procedures during the study period.

### 2.3. Statistical Analysis

Categorical variables are represented as frequencies and percentages. Continuous variables are presented as mean values ± standard deviation. Comparisons between continuous variables were performed using Student’s unpaired *t*-test. Grouped variables were compared using a two-way ANOVA. Survival analysis was carried out using the Kaplan–Meier procedure and differences in survival tested with the log-rank (Mantel–Cox) test. A *p* value < 0.05 was considered significant. Data analysis was conducted and performed in accordance with the guidelines of GraphPad Prism (Version 9.5.0 for macOS, GraphPad Software, San Diego, CA, USA, www.graphpad.com, accessed on 14 May 2023) and Microsoft Excel (Version 16.71, Microsoft Corporation (Redmond, WA, USA), 2023, available at: https://office.microsoft.com/excel, accessed on 14 May 2023). Sankey plots were created using open-source tool SANKEYMATIC by Steve Bogart (Naples, FL, USA), available at https://www.sankeymatic.com, accessed on 14 May 2023.

## 3. Results

### 3.1. Patient Cohort and Baseline Characteristics

From a total of 1407 patients who underwent TAVR between December 2011 and December 2022, 58 (4%) patients underwent valve-in-valve-procedures. Of these, 45 (78%) underwent a TiSAVR procedure, while 13 (22%) underwent a TiTAVR procedure. One patient underwent a third intervention and was assigned to the TiTAVR group in accordance with his last two interventions. In terms of previous interventions, most patients had undergone previous heart surgery (50 patients, 86%), including surgical aortic valve replacement (46 patients, 79%) and coronary artery bypass grafting (19 patients, 33%). Among the patients, 11 (19%) had a prior pacemaker, and 22 (38%) had undergone percutaneous coronary intervention. (Table 1) Patients undergoing valve-in-valve-procedures had significantly higher EuroSCORE II risk stratifications (8.6 ± 8.5 versus 3.4 ± 3.3, *p* < 0.001) and had undergone more previous interventions than TAVR patients. (86% versus 8%, *p* <0.001) (Supplemental Table S1). Patients undergoing TiTAVR were significantly older (83.7 ± 5.7 versus 77.6 ± 10 years, *p* = 0.015) and had more comorbidities than TiSAVR patients (Supplemental Table S2).

The ECG characteristics of the patients revealed that 23 (40%) had no changes, while 10 (17%) had right or left bundle branch block, 6 (10%) had any type of AV block, 18 (31%) had atrial fibrillation, and 4 (7%) had a paced rhythm. Pre-procedural transthoracic echocardiography (TTE) showed mean left ventricular ejection fraction (LVEF) of 51.0 ± 13.8%, mean aortic valve mean gradient of 31.0 ± 16.5 mmHg, mean aortic valve peak gradient of 50.8 ± 24.6 mmHg, and mean aortic valve area of 0.95 ± 0.46 cm^2^ (Table 2).

### 3.2. Incidence and Trends of Valve-in-Valve Procedures

**Figure 1:**
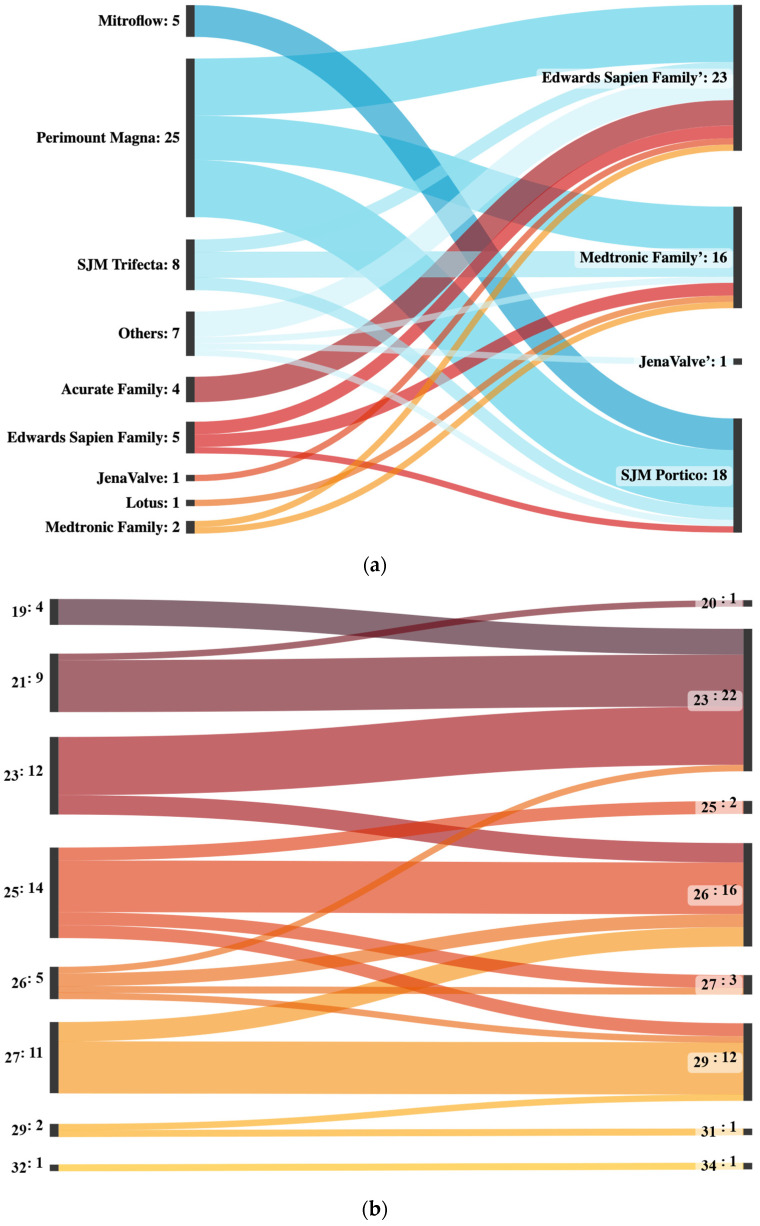

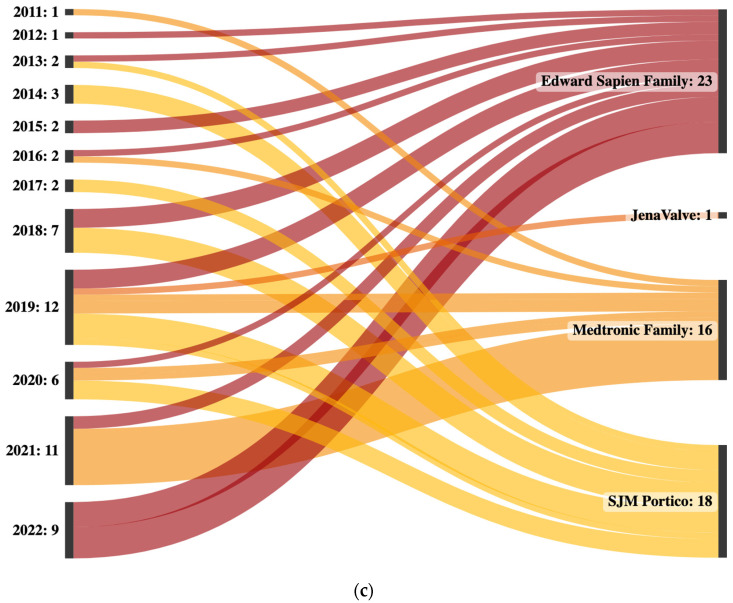
(**a**) **Sankey plot of all interventions with valves used in the index procedures on the left and valves in the valve-in-valve-intervention on the right.** Lines of index procedures in which surgical aortic valve replacements were performed are colored blue, while lines of index procedures in which transcatheter aortic valve implantations were performed are colored red. Acurate family includes Acurate neo and Acurate neo 2. Edwards Sapien family valves include Sapien XT, Sapien 3 and Sapien 3 Ultra. Medtronic family includes CoreValve, Evolut R, Evolut pro and Evolut pro+. The “Others” category includes Abbott Epic, Freedom Solo, Sulzer Vascutec and Toronto SPV. (**b**) **Sankey plot of all interventions sorted by initial valve size on the left and valve-in-valve sizes on the right.** All sizes are given in millimeters. (**c**) **Sankey plot of all interventions sorted by year and corresponding valve type.** Edwards Sapien family valves include Sapien XT, Sapien 3 and Sapient 3 Ultra. Medtronic family includes CoreValve, Evolut R and Evolut pro.

The most commonly used surgical index valves were Perimount Magna (25 patients), SJM Trifecta (8 patients) and Mitroflow (5 patients), while the most common interventional index valves were from the Edwards Sapien (5 patients) and Acurate families (4 patients) (Figure 1a).

The incidence of valve-in-valve-procedures increased from one in 2011 and 2012 to an average of ten in 2021 and 2022. Several different valve types were used in valve-in-valve-procedures from 2011 to 2022. The most commonly used valve types were from the Edwards Sapien family, which was used in a total of 23 procedures, including 9 in 2022 alone. The SJM Portico was the second most commonly used valve, with a total of 18 procedures, followed by Medtronic family with a total of 16 procedures. In 2019, a single JenaValve was implanted. In the initial years of the observational period, all implanted valve types were implanted equally often. Starting in 2021, there was less homogeneity, with mostly valves from the Medtronic family being implanted in 2021 and only valves from the Edwards Sapien family being implanted in 2022 (Supplemental Table S1 and Figure 1b).

### 3.3. Procedural Characteristics

**Figure 2:**
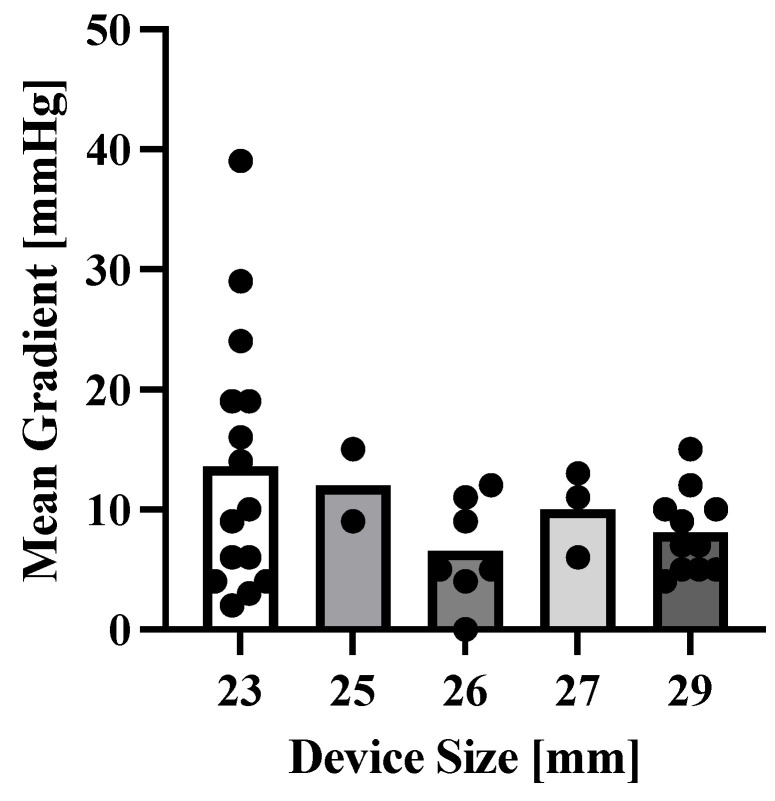
**Correlation between device size and mean gradient post-interventionally.** Data points represent all available mean gradient measurements post-interventionally. Bars represent the mean of all data points.

**Figure 3:**
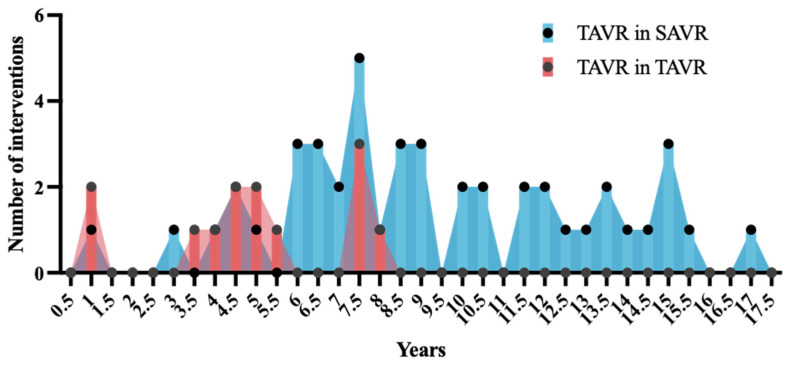
**Time between index and redo procedure** with transcatheter aortic valve replacement (TAVR) in TAVR shown in red and TAVR in surgical aortic valve replacement shown in blue.

Among the reasons for TiSAVR or TiTAVR, 28 (48%) of patients had severe prosthetic stenosis, 17 (29%) had severe prosthetic regurgitation, and 10 (17%) had combined prosthetic regurgitation and stenosis. Most patients (45, 78%) had previously undergone SAVR while the remaining 13 (22%) had prior TAVR. The most commonly used device size was 25 mm or less, which was used in 39 (67%) patients (Figure 1c). Among the new device types used in this study, self-expanding valves were used in 35 (60%) patients while balloon-expandable valves were used in 23 (40%) patients. No patient received a mechanically expanding valve. Pre-procedural invasive gradients had a mean of 27.2 ± 17.2 mmHg with a peak of 35.7 ± 26.1 mmHg. Postprocedural invasive gradients decreased significantly to a mean of 9.5 ± 7.6 mmHg with a peak of 10.7 ± 10.0 mmHg (*p* < 0.0001). Smaller valve size was neither significantly associated with higher gradients (*p* = 0.26), nor with a higher rate of adverse outcomes (29% versus 52%, *p* = 0.07) (Figure 2). The mean time between interventions was 8.6 ± 3.9 overall or 9.6 ± 3.8 years for TiSAVR and 5.3 ± 2.3 years for TiTAVR (*p* < 0.001) (Figure 3).

**Figure 4:**
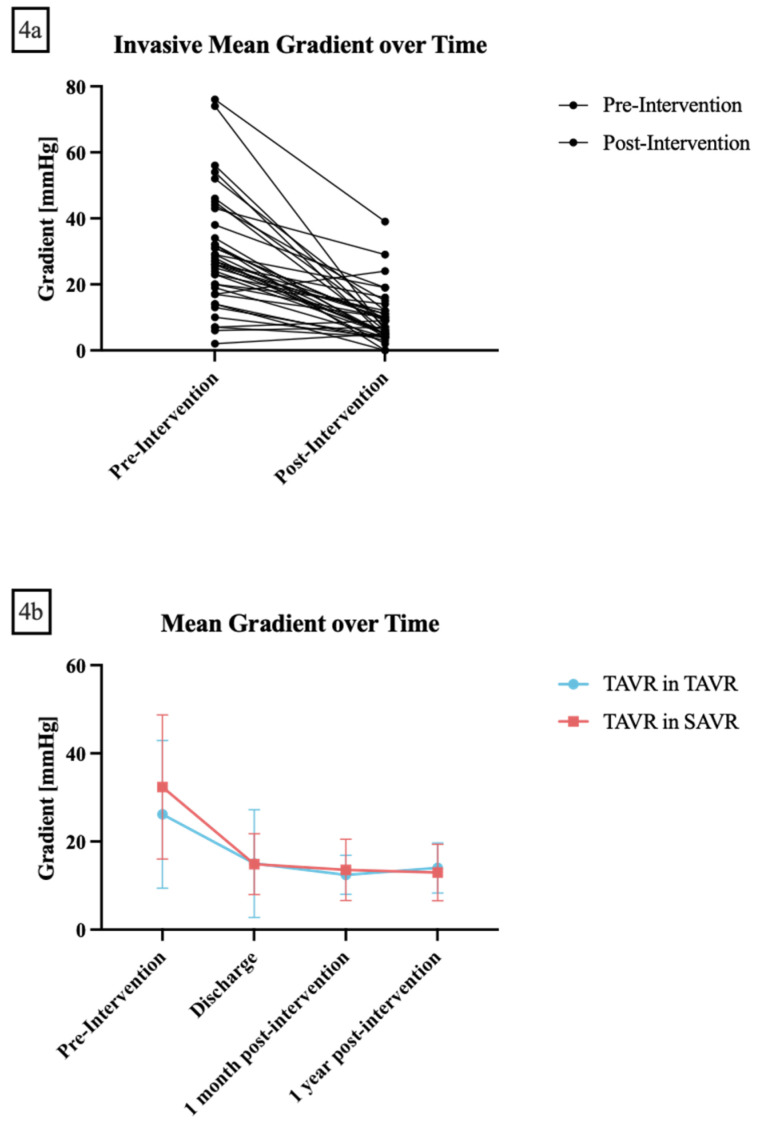
(**a**) **Invasive mean gradient before and after the intervention.** (**b**) **Trans-thoracal echocardiographic gradients before, at discharge, one month and one-year post-intervention.** Transcatheter aortic valve replacement (TAVR) in TAVR is shown in red while TAVR in surgical aortic valve replacement is shown in blue. Data are presented as mean ± standard deviation.

Post-procedural echocardiographic mean gradients at one month post-procedure were available for 34 patients and showed a mean of 13.5 ± 6.9 mmHg, while mean gradients at one year post-procedure were available for 21 patients and showed a mean of 13.2 ± 6.3 mmHg (Table 3, Figure 4a,b).

### 3.4. Procedural Outcomes

Technical success could be assessed in 57 patients (98%) and was achieved in 50 patients (88%) overall, with no difference between groups (TiSAVR 40 out of 45 patients (89%) versus TiTAVR 10 out of 12 patients (83%)) (*p* = 0.61). Device success could be assessed in 52 patients (90%) and was achieved in 44 patients (85%) overall. Device success could be achieved in 34 of 42 patients in the TiSAVR group (81%) and in 10 of 10 patients in the TiTAVR group (100%) (*p* = 0.19).

Of the patients, 39 (67%) did not experience any procedural or post-procedural complication. One patient (2%) died during the procedure with an already reduced pre-interventional LVEF of 25% without any bleeding or relevant cardiac tamponade apparent in transthoracic echocardiography (TTE), while two others (4%) died while in the hospital. The first of the two had a difficult post-procedural course with acute respiratory distress syndrome and multiple infarctions in CT and died 11 days post-interventionally. The second came to the hospital due to severely decompensated heart failure, received an emergency valve-in-valve procedure into the degenerated index valve and died of multiple organ failure eight days after the procedure. Two patients (4%) experienced valve embolization. In both patients, the valve could not be deployed correctly, embolized in the direction of the ventricle and required conversion to open-heart surgery in one of the two. In the other patient, the device could be pulled back and replaced with another one. No cases of coronary occlusion or myocardial infarction were reported. Thrombocyte count and hemoglobin were comparable in those with or without adverse outcomes (177.0 versus 201.5 g/L (10^9), *p* = 0.26 and 125.3 versus 114.8 g/L, *p* = 0.07). No patient had been diagnosed with valve thrombosis or systemic embolization (Table 4). Patients receiving a balloon-expandable valve in a degenerated valve had a similar complication rate to recipients of self-expandable valves (0.40 versus 0.43%, *p* = 0.80).

**Figure 5:**
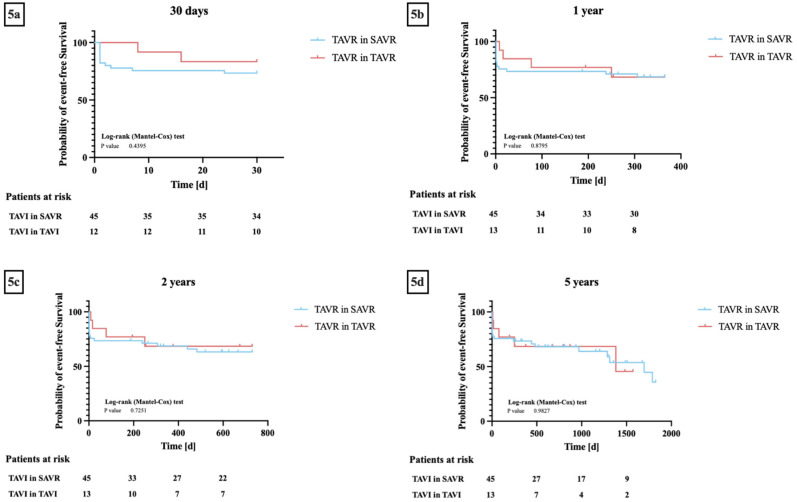
(**a**–**d**) Kaplan–Meier curves for event-free survival* between transcatheter aortic valve replacement (TAVR) in previous surgical aortic valve replacement (TiSAVR) and TAVR in TAVR (TiTAVR): (**a**) 30 days, (**b**) one year, (**c**) two years, (**d**) five years. * Event-free survival as defined by Valve Academic Research Consortium 3. Log-rank testing showed no significant differences in event-free survival between the two groups at all follow-up timepoints.

Kaplan–Meier analyses regarding event-free survival were carried out between patients from the TiSAVR and TiTAVR groups at 30 days, one year, two years, and five years after the procedure. At 30 days, the incidence of endpoints defined by VARC-3 was 24% in the TiSAVR group and 17% in the TiTAVR group (*p* = 0.48). At one year, the incidence in the TiSAVR group was 31% and 33% in the TiTAVR group (*p* = 0.98). At two years, the incidence in the TiSAVR group was 36% and 33% in the TiTAVR group (*p* = 0.75). Finally, at five years, the incidence in the TiSAVR group was 42% and 42% in the TiTAVR group (*p* = 0.81). The log-rank test showed no significant differences between the two groups in terms of overall event-free survival rates and incidence of clinical endpoints defined by VARC-3 at any follow-up time point (*p* = 0.44, *p* = 0.88, *p* = 0.73, *p* = 0.98) (Figure 5).

**Figure 6:**
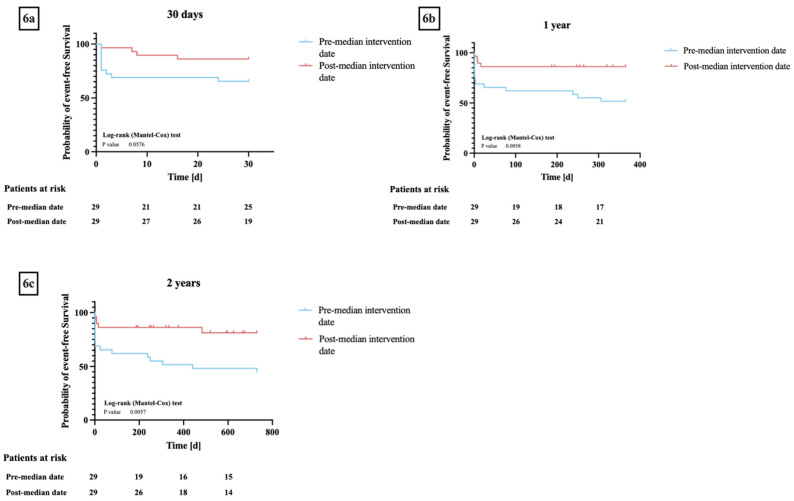
(**a**–**c**) Kaplan–Meier curves for event-free survival* between groups pre and post the median valve-in-valve-procedure date: (**a**) 30 days, (**b**) one year, (**c**) two years. * Event-free survival as defined by Valve Academic Research Consortium 3. Log-rank testing showed no significant differences in event-free survival between the two groups at 30 days (*p* = 0.06) but was able to show significant differences in 1- and 2-year event-free survival (*p* < 0.01).

Patients who underwent a valve-in-valve procedure before December 2019 had lower rates of procedural (25 patients (86%) versus 26 patients (90%), *p* = 0.73) and technical success (12 patients (63%) versus 14 patients (82%), *p* = 0.21) and lower 30-day (19 patients (66%) versus 25 patients (86%), *p* = 0.06) and one-year (15 patients (52%) versus 25 patients (86%), *p* < 0.01) event-free survival compared to patients who underwent the procedure after December 2019 (Figure 6a–c).

## 4. Discussion

In this study, we aimed to provide an overview of valve-in-valve procedures over a period of eleven years in a tertiary university center. Our findings contribute to the current body of evidence on valve-in-valve interventions, particularly in the context of an increasing number of patients receiving TAVR at younger ages. We report five major findings:

First, in this real-world cohort of TAVR patients consecutively recruited over 10 years, a valve-in-valve procedure was performed in 58 out of 1407 procedures (4%). Between 2011 and 2022, a steady increase in valve-in-valve procedures was observed in both groups, and particularly in the TiTAVR group in recent years. Exemplarily, half of all valve-in-valve procedures of the ten-year observational period were performed between December 2021 and December 2022. This trend aligns with the increasing utilization of TAVR in younger patients, as opposed to TAVR being restricted to only high-risk or inoperable patients at the start of the registry. In addition, techniques and approval from authorities for certain devices for valve-in-valve procedures have evolved in recent years [15]. The average time between the first and second intervention was 8.6 years in the overall cohort, whereas TiTAVR was performed earlier than TiSAVR (5.3 ± 2.3 vs 9.6 ± 3.8 years). This observation may imply a potentially earlier degeneration of TAVR valves. However, TiTAVR patients were significantly older (85 versus 79 years, *p* = 0.015), and younger patients with degenerated SAVR valves may have undergone redo-SAVR, which was not included in the scope of our registry.

Second, technical success was achieved in 88% and device success in 85% of patients overall. The most common complications were minor vascular complications in 9% of patients followed by permanent pacemaker implantation in 7% of patients, half of which presented with pre-interventional ECG abnormalities. There was no difference between TiSAVR and TiTAVR in terms of technical or device success (89% versus 83%, *p* = 0.61 and 81% versus 100%, *p* = 0.19) Balloon- and self-expandable valve-in-valve interventions exhibited comparable rates of complications (0.40% versus 0.43%, *p* = 0.80). Nevertheless, it is important to note that this finding may be influenced by a selection bias.

Third, while the number of procedures has been steadily rising, a contrasting trend has emerged in relation to complications. Prior to December 2021 (as the median for all procedures), 86% of patients achieved technical success, while device success was reported in 63% of patients. In subsequent cases, the rates of technical and device success improved, reaching 90% and 82% of patients, respectively (*p* = 0.73 and *p* = 0.21).

Fourth, our study demonstrated event incidences of 24% after one month and 33% at one year, encompassing a wide range of categories of events as defined in VARC-3. These events encompassed procedural or in-hospital death, coronary occlusion, stroke, permanent pacemaker implantation, myocardial infarction, major (stent or surgery) vascular complications, major bleeding requiring more than two transfusions, and valve embolization. The occurrence of these events aligned with prior reports and was expected, given the high-risk cohort with a mean EuroSCORE II of 8.7%. Furthermore, incidences were comparable in both groups, indicating similarity of procedure-related risks regardless of the type of index valve (33% in TiSAVR, 31% in TiTAVR at one year).

Fifth, we observed significant improvements in mean gradients after the procedure compared to pre-procedure values with median deltas between pre- and post-procedure of 20 mmHg in TiSAVR and 14 mmHg in TiTAVR, respectively. In addition, gradients remained stable at one-month and one-year follow-up, indicating successful and durable alleviation of valve stenosis or regurgitation by the valve-in-valve approach. These results are consistent with prior studies demonstrating favorable hemodynamic outcomes following valve-in-valve procedures [16].

This study analyzing real world data of valve-in-valve procedures in a tertiary university hospital over a time frame of 10 years corroborates and extends prior findings in the field that have been reported. The number of patients with late degeneration of bioprosthetic valves is bound to increase in the future. Explantation of these failed valves is a challenging procedure, as a result of comorbidities, advancing age, anatomical factors, tissue ingrowth and valve design [17]. In younger low-risk patients, the repeatability of TAVR will be an increasingly important issue [18]. Recent data from 37 centers involved in the Redo-TAVR registry have shown feasibility and safety of redo-TAVR, with acceptable outcomes in 212 patients. Coronary obstruction occurred in two patients and no patient died during the redo-TAVR procedure [19]. To date, four significant multi-center retrospective registry studies have been reported with 30-day mortality <5% in all four studies, and 12-month mortality between 10.0% and 15.6% [7,20,21,22], whereas redo-SAVR was associated with higher mortality, procedural complications and length of hospital stay [23,24]. Notably, within our cohort, we observed an absence of acute coronary occlusions, despite previous evidence indicating their occurrence in approximately 3.5% of interventions [25]. Preventing coronary obstructions remains a significant objective in valve-in-valve cases. Pre-procedural computed tomography (CT) is necessary before a valve-in-valve procedure to assess the completeness of initial valve expansion, the index valve internal diameter and the relationships between the failed valve and aortic root anatomy, including the coronary ostia, sinus width and the sinotubular junction. Strategies to optimize repeatability may include thoughtful selection of the first valve implanted in younger patients, in whom repeatability may be of concern [18]. Permanent pacemaker implantation is an ongoing issue in native as well as in valve-in-valve procedures. The permanent pacemaker implantation rate was 7% with no difference between groups. Notably, it was lower compared to the rate in the national SWISS TAVI registry in native aortic annuli (18%) [26]. Sá et al. also demonstrated lower rates of pacemaker implantation in patients who underwent TiTAVR versus TiSAVR among 16207 patients from 12 studies, whereas Raschpichler et al. found similar rates in both groups in a meta-analysis with 8881 patients included in 15 studies [23,27].

Screening for acute infections is crucial in all patients undergoing TAVR and especially valve-in-valve TAVR to ensure optimal safety and outcomes [12]. Patients undergoing valve-in-valve procedures should furthermore be screened for thrombophilia, as valve thrombosis is a potentially lethal complication, especially in recipients of two bioprosthetic valves [28].

At 30 days, 8% of patients had a mean gradient of >20 mmHg, which was lower compared to similar cohorts. Raschpichler reported permanent pacemaker implantation rates in around 26.8% to 37.0% of patients following valve-in-valve procedures, which was associated with increased mortality at one year [16,29]. Our study is one of the first which reports gradients in TiSAVR and TiTAVR and finds no relevant difference between groups. Several retro- and prospective registries have suggested a lower peri-procedural mortality and morbidity in patients undergoing valve-in-valve interventions compared to Redo-SAVR [9,10,16,24].

So far, no prospective studies in the field of TiTAVR have been performed. An upcoming study (NCT05601453) will include approximately 200 patients for TiTAVR with the SAPIEN 3/Ultra valve in 50 high-volume European TAVR centers. The aim of this study is to define patient-related and procedural factors predicting procedural results and clinical outcomes [22].

Despite the significant findings of our study, there are some limitations that should be acknowledged. First, our study was conducted in a single tertiary center, which may have led to selection biases and limit the generalizability of the results. Future studies with larger sample sizes and multi-center collaborations would be valuable to further extend our findings. Second, although the follow-up duration in our study was comparably longer than in the current literature, longer-term outcomes of valve-in-valve procedures should be investigated in future studies.

In conclusion, our study provides insights into the incidence, trends, and long-term outcomes of valve-in-valve procedures over the last decade, highlighting the increasing utilization of valve-in-valve interventions, particularly in the context of TiTAVR procedures as well as comparable clinical outcomes and echocardiographic gradients among TiSAVR and TiTAVR patients. Further research is warranted, to optimize procedural success rates and investigate the long-term durability of valve-in-valve interventions in prospective, international trials.

## Figures and Tables

**Table 1:** Patient baseline characteristics.

**Basic Characteristics**	**Patients (N = 58)**
Female, n (%)	26 (45%)
Age, years	79 ± 9.4
Body mass index, kg/m^2^	26.8 ± 5.5
LVEF, %	51.0 ± 13.8
EuroSCORE, %	8.7 ± 8.5
**Preconditions**	**Patients (N = 58)**
Diabetes, n (%)	21 (36%)
Dyslipidemia, n (%)	35 (60%)
Hypertension, n (%)	47 (81%)
Coronary artery disease, n (%)	38 (66%)
Myocardial infarction, n (%)	14 (24%)
eGFR (CKD-EPI), mL/min	50.1 ± 24.1
Hemoglobin, g/L	121.0 ± 22.0
**Previous Interventions, n (%)**	**Patients (N = 58)**
Any heart surgery	50 (86%)
Surgical aortic valve replacement	46 (79%)
Coronary artery bypass grafting	19 (33%)
Transcatheter aortic valve replacement	13 (22%)
Prior pacemaker	11 (19%)
Percutaneous coronary intervention	22 (38%)

Values are given as mean ± SD or n (%). LVEF = left ventricular ejection fraction; EuroSCORE = European System for Cardiac Operative Risk Evaluation; eGFR = estimated glomerular filtration rate.

**Table 2:** Patient electrocardiogram and echocardiography characteristics.

**ECG Characteristics, n (%)**	**Patients (N = 58)**
No changes	23 (40%)
Right or left bundle branch block	10 (17%)
Any AV block	6 (10%)
Atrial fibrillation	18 (31%)
Paced rhythm	4 (7%)
**Echocardiography Parameters**	**Patients (N = 46)**
LVEF, %	51.0 ± 13.8
Aortic valve mean gradient, mmHg	31.0 ± 16.5
Aortic valve peak gradient, mmHg	50.8 ± 24.6
Aortic valve area, cm^2^	0.95 ± 0.46

Values are given as mean ± SD or n (%). ECG = electrocardiogram; AV = atrioventricular; LVEF = left ventricular ejection fraction.

**Table 3:** Procedural characteristics.

**Reason for ViV-TAVI, n (%)**	**Patients (N = 58)**
Prosthetic regurgitation	
≤Mild	0 (0%)
>Mild	17 (29%)
Prosthetic stenosis	28 (48%)
Combined regurgitation and stenosis	10 (17%)
**Old device Type, n (%)**	**Patients (N = 58)**
SAVR	45 (78%)
TAVI	13 (22%)
Device size	
≤25 mm	39 (67%)
>25 mm	19 (33%)
**New Device Type, n (%)**	**Patients (N = 58)**
Balloon-expandable	23 (40%)
Self-expanding	35 (60%)
Mechanically expanding	0 (0%)
Device size	
≤25 mm	33 (57%)
>25 mm	25 (43%)
**Procedural Measurements**	**Patients (N = 46)**
Invasive gradients (mmHg)	
Preprocedural mean	27.2 ± 17.2
Preprocedural peak	35.7 ± 26.1
Postprocedural mean	9.5 ± 7.6
Postprocedural peak	10.7 ± 10.0
Time between valve interventions (years)	8.6 ± 3.9
**Post-Procedural Echo Gradients (mmHg)**	**Patients**
1 month post-procedural, n	13.3 ± 6.4, 37
1 year post-procedural, n	13.2 ± 6.3, 21

Values are given as mean ± SD or n (%). ViV-TAVI = valve-in-valve transcatheter aortic valve implantation, SAVR = surgical aortic valve replacement.

**Table 4:** Patient procedural and post-procedural complications.

Complications, n (%)	Patients (N = 58)
No complications	39 (67%)
Death	
Procedural death	1 (2%)
Procedural reanimation	4 (7%)
In-hospital death	2 (4%)
Coronary occlusion	0 (0%)
Stroke	0 (0%)
Transient ischemic attack	1 (2%)
Permanent pace maker	4 (7%)
Myocardial infarction	0 (0%)
Vascular complication	
Major complication (stent or surgery)	3(5%)
Minor complication (compression, ballooning, blood transfusion)	5(9%)
Valve embolization	2 (4%)

Values are given as mean ± SD or n (%).

## Data Availability

The data presented in this study are available on request from the corresponding author. The data are not publicly available due to privacy.

## Supplementary Materials

The following supporting information can be downloaded at: https://www.mdpi.com/article/10.3390/jcm12185868/s1, Table S1: Patient Baseline Characteristics compared between patients undergoing valve-in-valve- (ViV) procedures and the control group. Table S2: Patient Baseline Characteristics compared between patients undergoing TiSAVR and TiTAVR.

## Abbreviations

AS	aortic stenosis
CT	computed tomography
ECG	electrocardiogram
eGFR	estimated glomerular filtration rate
EuroSCORE	European System for Cardiac Operative Risk Evaluation
LVEF	left ventricular ejection fraction
SAVR	surgical aortic valve replacement
TAVR	transcatheter aortic valve replacement
TiSAVR	TAVR in SAVR
TiTAVR	TAVR in TAVR
TTE	transthoracic echocardiography
VARC	Valve Academic Research Consortium

## References

[1] Otto C.M., Nishimura R.A., Bonow R.O., Carabello B.A., Erwin J.P., Gentile F., Jneid H., Krieger E.V., Mack M., McLeod C. (2021). 2020 ACC/AHA Guideline for the Management of Patients With Valvular Heart Disease: A Report of the American College of Cardiology/American Heart Association Joint Committee on Clinical Practice Guidelines. Circulation.

[2] Vahanian A., Beyersdorf F., Praz F., Milojevic M., Baldus S., Bauersachs J., Capodanno D., Conradi L., De Bonis M., De Paulis R. (2022). 2021 ESC/EACTS Guidelines for the management of valvular heart disease. Eur. Heart J..

[3] Kundi H., Strom J.B., Valsdottir L.R., Elmariah S., Popma J.J., Shen C., Yeh R.W. (2018). Trends in Isolated Surgical Aortic Valve Replacement According to Hospital-Based Transcatheter Aortic Valve Replacement Volumes. JACC Cardiovasc. Interv..

[4] Sharma T., Krishnan A.M., Lahoud R., Polomsky M., Dauerman H.L. (2022). National Trends in TAVR and SAVR for Patients With Severe Isolated Aortic Stenosis. J. Am. Coll. Cardiol..

[5] Toff W.D., Hildick-Smith D., Kovac J., Mullen M.J., Wendler O., Mansouri A., Rombach I., Abrams K.R., Conroy S.P., UK TAVI Trial Investigators (2022). Effect of Transcatheter Aortic Valve Implantation vs Surgical Aortic Valve Replacement on All-Cause Mortality in Patients with Aortic Stenosis: A Randomized Clinical Trial. JAMA.

[6] Bapat V.N., Zaid S., Fukuhara S., Saha S., Vitanova K., Kiefer P., Squiers J.J., Voisine P., Pirelli L., von Ballmoos M.W. (2021). Surgical Explantation After TAVR Failure: Mid-Term Outcomes From the EXPLANT-TAVR International Registry. JACC Cardiovasc. Interv..

[7] Tang G.H.L., Zaid S., Kleiman N.S., Goel S.S., Fukuhara S., Marin-Cuartas M., Kiefer P., Abdel-Wahab M., De Backer O., Søndergaard L. (2023). Explant vs Redo-TAVR After Transcatheter Valve Failure: Mid-Term Outcomes From the EXPLANTORREDO-TAVR International Registry. JACC Cardiovasc. Interv..

[8] Al-Abcha A., Saleh Y., Boumegouas M., Prasad R., Herzallah K., Baloch Z.Q., Abdelkarim O., Rayamajhi S., Abela G.S. (2021). Meta-Analysis of Valve-in-Valve Transcatheter Aortic Valve Implantation Versus Redo-surgical Aortic Valve Replacement in Failed Bioprosthetic Aortic Valve. Am. J. Cardiol..

[9] Spaziano M., Mylotte D., Thériault-Lauzier P., De Backer O., Søndergaard L., Bosmans J., Debry N., Modine T., Barbanti M., Tamburino C. (2017). Transcatheter aortic valve implantation versus redo surgery for failing surgical aortic bioprostheses: A multicentre propensity score analysis. EuroIntervention.

[10] Gatta F., Haqzad Y., Gradinariu G., Malvindi P.G., Khalid Z., Suelo-Calanao R.L., Moawad N., Bashir A., Rogers L.J., Lloyd C. (2023). Redo aortic valve replacement vs valve-in-valve trans-catheter aortic valve implantation: A UK propensity-matched analysis. Monaldi Arch. Chest Dis..

[11] Pilgrim T., Franzone A., Stortecky S., Nietlispach F., Haynes A.G., Tueller D., Toggweiler S., Muller O., Ferrari E., Noble S. (2017). Predicting Mortality After Transcatheter Aortic Valve Replacement: External Validation of the Transcatheter Valve Therapy Registry Model. Circ. Cardiovasc. Interv..

[12] Stortecky S., Heg D., Tueller D., Pilgrim T., Muller O., Noble S., Jeger R., Toggweiler S., Ferrari E., Taramasso M. (2020). Infective Endocarditis After Transcatheter Aortic Valve Replacement. J. Am. Coll. Cardiol..

[13] Attinger-Toller A., Ferrari E., Tueller D., Templin C., Muller O., Nietlispach F., Toggweiler S., Noble S., Roffi M., Jeger R. (2021). Age-Related Outcomes After Transcatheter Aortic Valve Replacement: Insights From the SwissTAVI Registry. JACC Cardiovasc. Interv..

[14] Généreux P., Piazza N., Alu M.C., Nazif T., Hahn R.T., Pibarot P., Bax J.J., Leipsic J.A., Blanke P., VARC-3 WRITING COMMITTEE (2021). Valve Academic Research Consortium 3: Updated endpoint definitions for aortic valve clinical research. Eur. Heart J..

[15] Nalluri N., Atti V., Munir A.B., Karam B., Patel N.J., Kumar V., Vemula P., Edla S., Asti D., Paturu A. (2018). Valve in valve transcatheter aortic valve implantation (ViV-TAVI) versus redo-Surgical aortic valve replacement (redo-SAVR): A systematic review and meta-analysis. J. Interv. Cardiol..

[16] Raschpichler M.C., Woitek F., Chakravarty T., Flint N., Yoon S.-H., Mangner N., Patel C.G., Singh C., Kashif M., Kiefer P. (2020). Valve-in-Valve for Degenerated Transcatheter Aortic Valve Replacement Versus Valve-in-Valve for Degenerated Surgical Aortic Bioprostheses: A 3-Center Comparison of Hemodynamic and 1-Year Outcome. J. Am. Heart Assoc..

[17] Fukuhara S., Brescia A.A., Shiomi S., Rosati C.M., Yang B., Kim K.M., Deeb G.M. (2021). Surgical explantation of transcatheter aortic bioprostheses: Results and clinical implications. J. Thorac. Cardiovasc. Surg..

[18] Webb J.G., Blanke P., Meier D., Sathananthan J., Lauck S., Chatfield A.G., Jelisejevas J., Wood D.A., Akodad M. (2022). TAVI in 2022: Remaining issues and future direction. Arch. Cardiovasc. Dis..

[19] Landes U., Webb J.G., De Backer O., Sondergaard L., Abdel-Wahab M., Crusius L., Kim W.-K., Hamm C., Buzzatti N., Montorfano M. (2020). Repeat Transcatheter Aortic Valve Replacement for Transcatheter Prosthesis Dysfunction. J. Am. Coll. Cardiol..

[20] Landes U., Sathananthan J., Witberg G., De Backer O., Sondergaard L., Abdel-Wahab M., Holzhey D., Kim W.-K., Hamm C., Buzzatti N. (2021). Transcatheter Replacement of Transcatheter Versus Surgically Implanted Aortic Valve Bioprostheses. J. Am. Coll. Cardiol..

[21] Makkar R.R., Kapadia S., Chakravarty T., Cubeddu R., Mahoney P., Yadav P., Iyer P., Kaneko Y., Kodali S., Mack M. Outcomes of Repeat TAVR with Balloon-Expandable SAPIEN 3/Ultra Valves. tctMD. https://www.tctmd.com/slide/outcomes-repeat-tavr-balloon-expandable-sapien-3ultra-valves.

[22] Tarantini G., Delgado V., de Backer O., Sathananthan J., Treede H., Saia F., Blackman D., Parma R. (2023). Redo-Transcatheter Aortic Valve Implantation Using the SAPIEN 3/Ultra Transcatheter Heart Valves-Expert Consensus on Procedural Planning and Techniques. Am. J. Cardiol..

[23] Sá M.P.B.O., Van den Eynde J., Simonato M., Cavalcanti L.R.P., Doulamis I.P., Weixler V., Kampaktsis P.N., Gallo M., Laforgia P.L., Zhigalov K. (2021). Valve-in-Valve Transcatheter Aortic Valve Replacement Versus Redo Surgical Aortic Valve Replacement: An Updated Meta-Analysis. JACC Cardiovasc. Interv..

[24] Thandra A., Abusnina W., Jhand A., Shaikh K., Bansal R., Pajjuru V.S., Al-Abdouh A., Kanmanthareddy A., Alla V.M. (2021). Valve-in-valve transcatheter aortic valve replacement versus redo surgical valve replacement for degenerated bioprosthetic aortic valve: An updated meta-analysis comparing midterm outcomes. Catheter. Cardiovasc. Interv..

[25] Dvir D., Leipsic J., Blanke P., Ribeiro H.B., Kornowski R., Pichard A., Rodés-Cabau J., Wood D.A., Stub D., Ben-Dor I. (2015). Coronary obstruction in transcatheter aortic valve-in-valve implantation: Preprocedural evaluation, device selection, protection, and treatment. Circ. Cardiovasc. Interv..

[26] Ferrari E., Stortecky S., Heg D., Muller O., Nietlispach F., Tueller D., Toggweiler S., Noble S., Maisano F., Roffi M. (2019). The hospital results and 1-year outcomes of transcatheter aortic valve-in-valve procedures and transcatheter aortic valve implantations in the native valves: The results from the Swiss-TAVI Registry. Eur. J. Cardiothorac. Surg..

[27] Raschpichler M., de Waha S., Holzhey D., Schwarzer G., Flint N., Kaewkes D., Bräuchle P.T., Dvir D., Makkar R., Ailawadi G. (2022). Valve-in-Valve Transcatheter Aortic Valve Replacement Versus Redo Surgical Aortic Valve Replacement for Failed Surgical Aortic Bioprostheses: A Systematic Review and Meta-Analysis. J. Am. Heart Assoc..

[28] Calabrò P., Gragnano F., Niccoli G., Marcucci R., Zimarino M., Spaccarotella C., Renda G., Patti G., Andò G., Moscarella E. (2021). Antithrombotic Therapy in Patients Undergoing Transcatheter Interventions for Structural Heart Disease. Circulation.

[29] Webb J.G., Mack M.J., White J.M., Dvir D., Blanke P., Herrmann H.C., Leipsic J., Kodali S.K., Makkar R., Miller D.C. (2017). Transcatheter Aortic Valve Implantation Within Degenerated Aortic Surgical Bioprostheses: PARTNER 2 Valve-in-Valve Registry. J. Am. Coll. Cardiol..

