# Supplementary material for: Incidence and Outcomes of Valve-in-Valve Transcatheter Aortic Valve Implantation in Failed Bioprosthetic Valves
Source: J Clin Med. 2023 Sep 9;12(18):5868. doi: 10.3390/jcm12185868 (PMC10531770; doi:10.3390/jcm12185868)
Supplement: Supplementary file 1 [file jcm-12-05868-s001.zip › jcm-2516826-supplementary.pdf]

Supplementary Table S1. Patient Baseline Characteristics compared between patients undergoing valve-in-valve- (ViV) procedures and the control group.

Variable	N	Overall, N = 1,370[1]	ViV, N = 58[1]	Control, N = 1,312[1]	p-value[2]
**Basic characteristics**					
Sex	1,365				0.4
Male		684 (50%)	32 (55%)	652 (50%)	
Female		681 (50%)	26 (45%)	655 (50%)	
Age	1,364	82.7 (78.9, 86.2)	80.0 (76.0, 84.0)	82.7 (79.0, 86.3)	0.004
BMI	1,361	26.2 (23.5, 29.4)	25.0 (23.0, 29.0)	26.2 (23.5, 29.4)	0.4
LVEF	1,265	58 (45, 62)	54 (44, 60)	59 (45, 62)	0.094
Euro SCORE II	1,178	2.13 (1.26, 4.14)	6.24 (3.00, 11.00)	2.03 (1.20, 3.90)	<0.001
**Preconditions**					
Diabetes	1,365	395 (29%)	21 (36%)	374 (29%)	0.2
Dyslipidemia	1,365	809 (59%)	35 (60%)	774 (59%)	0.9
Hypertension	1,365	1,110 (81%)	47 (81%)	1,063 (81%)	>0.9
Coronary artery disease	1,365	786 (58%)	38 (66%)	748 (57%)	0.2
Myocardial infarction	1,365	239 (18%)	14 (24%)	225 (17%)	0.2
eGFR	1,358	51 (38, 66)	49 (35, 63)	51 (38, 66)	0.3
Hemoglobin	1,361	126 (114, 137)	120 (108, 138)	126 (115, 137)	0.11
**Previous intereventions**					
Any Heart Surgery	1,365	152 (11%)	50 (86%)	102 (7.8%)	<0.001
Surgical Aortic Valve Replacement	1,370	56 (4.1%)	46 (79%)	10 (0.8%)	<0.001
Coronary Artery Bypass Grafting	1,370	113 (8.2%)	19 (33%)	94 (7.2%)	<0.001
Prior pacemaker	1,365	149 (11%)	11 (19%)	138 (11%)	0.045
Percutaneous Coronary Intervention	1,365	487 (36%)	22 (38%)	465 (36%)	0.7

[1] n (%); Median (IQR)
[2] Pearson’s Chi-squared test; Wilcoxon rank sum test; Fisher’s exact test;
LVEF = Left ventricular ejection fraction, EUROSCORE = European system for cardiac operative risk evaluation; eGFR = estimated glomerular filtration rate

Supplementary Table S2. Patient Baseline Characteristics compared between patients undergoing TiSAVR and TiTAVR.

Variable	N	Overall, N = 58[1]	TiTAVR, N = 13[1]	TiSAVR, N = 45[1]	p-value[2]
**Basic characteristics**					
Sex	58				>0.9
Male		32 (55%)	7 (54%)	25 (56%)	
Female		26 (45%)	6 (46%)	20 (44%)	
Age	57	80 (76, 84)	85 (79, 87)	79 (74, 83)	0.015
BMI	57	25.0 (23.0, 29.0)	25.0 (22.0, 28.0)	26.0 (24.0, 29.0)	0.5
LVEF	56	54 (44, 60)	50 (45, 60)	55 (43, 60)	0.5
Euro SCORE II	58	6 (3, 11)	8 (4, 13)	6 (3, 9)	0.4
**Preconditions**					
Diabetes	58	21 (36%)	5 (38%)	16 (36%)	>0.9
Dyslipidemia	58	35 (60%)	9 (69%)	26 (58%)	0.5
Hypertension	58	47 (81%)	12 (92%)	35 (78%)	0.4
Coronary artery disease	58	38 (66%)	11 (85%)	27 (60%)	0.2
Myocardial infarction	58	14 (24%)	4 (31%)	10 (22%)	0.7
eGFR	58	49 (35, 63)	39 (31, 44)	52 (38, 69)	0.028
Hemoglobin	58	120 (108, 138)	108 (87, 117)	124 (113, 140)	0.008
**Previous intereventions**					
Any Heart Surgery	58	50 (86%)	5 (38%)	45 (100%)	<0.001
Surgical Aortic Valve Replacement	58	46 (79%)	1 (7%)	45 (100%)	<0.001
Coronary Artery Bypass Grafting	58	19 (33%)	2 (15%)	17 (38%)	0.2
Prior pacemaker	58	11 (19%)	3 (23%)	8 (18%)	0.7
Percutaneous Coronary Intervention	58	22 (38%)	7 (54%)	15 (33%)	0.2

[1] n (%); Median (IQR)
[2] Pearson's Chi-squared test; Wilcoxon rank sum test; Fisher's exact test;
TiSAVR = Transcatheter aortic valve replacement (TAVR) in surgical aortic valve replacement; TiTAVR = TAVR in TAVR; LVEF = Left ventricular ejection fraction, EUROSCORE = European system for cardiac operative risk evaluation; eGFR = estimated glomerular filtration rate;
